# Co-infection with *Bartonella bacilliformis* and *Mycobacterium* spp. in a coastal region of Peru

**DOI:** 10.1186/s13104-017-2977-y

**Published:** 2017-12-01

**Authors:** Wilmer Silva-Caso, Fernando Mazulis, Claudia Weilg, Miguel Angel Aguilar-Luis, Isabel Sandoval, German Correa-Nuñez, Dongmei Li, Xiuping Song, Qiyong Liu, Juana del Valle-Mendoza

**Affiliations:** 1 School of Medicine, Research and Innovation Centre of the Faculty of Health Sciences, Universidad Peruana de Ciencias Aplicadas, Av. San Marcos cuadra. 2, Chorrillos, Lima, Peru; 2 Laboratorio de Biología Molecular, Instituto de Investigación Nutricional, Lima, Peru; 3 Red de Salud Morropón-Chulucanas, Piura, Peru; 4 Departamento Académico de Ciencias Básicas, Universidad Nacional Amazónica de Madre de Dios, Madre de Dios, Peru; 5 State Key Laboratory for Infectious Disease Prevention and Control, Collaborative Innovation Center for Diagnosis and Treatment of Infectious Diseases, National Institute for Communicable Disease Control and Prevention, Chinese Center for Disease Control and Prevention, Changping, Beijing, China

**Keywords:** *Bartonella bacilliformis*, Carrions disease, *Mycobacterium*, Peru, PCR

## Abstract

**Objective:**

This study investigated an outbreak of Bartonellosis in a coastal region in Peru.

**Results:**

A total of 70 (n = 70) samples with clinical criteria for the acute phase of Bartonellosis and a positive peripheral blood smear were included. 22.85% (n = 16) cases of the samples were positive for *Bartonella bacilliformis* by PCR and automatic sequencing. Of those positive samples, 62.5% (n = 10) cases were positive only for *B. bacilliformis* and 37.5% (n = 6) cases were positive to both *Mycobacterium* spp. and *B. bacilliformis*. The symptom frequencies were similar in patients diagnosed with Carrion’s disease and those co-infected with *Mycobacterium* spp. The most common symptoms were headaches, followed by malaise and arthralgia.

## Introduction

Carrion’s disease, also known as Bartonellosis, is an emerging and forgotten disease caused by *Bartonella bacilliformis*, a gram-negative, pleomorphic, facultative intracellular bacteria that infects humans and has been responsible for over 100,000 deaths since its initial description [1, 2]. It’s main route of transmission is through the bite of phlebotomine sand flies, particularly *Lutzomyia verrucarum*, and other vectors such as *L. Peruensis*, *L. maramonensis* and *L. robusta* [3].

Bartonellosis is endemic to inter-Andean valleys located at altitudes between 500 and 3200 m above sea level (m.a.s.l.). Bartonellosis is endemic to certain regions in Peru and neighboring South American countries within valleys with similar altitudes and conditions [1, 4]. To date, very few cases of Carrion’s disease have been reported in lower altitude coastal territories [5].

Carrion’s disease has two clinical phases: Oroya’s fever and the Peruvian Wart [2]. The acute phase, also known as the hematic phase or Oroya’s fever, is clinically characterized by fever, headache, myalgias, bone pain and variable levels of anemia [6]. The pathogenicity of the bacteria includes a massive intraerythrocytic invasion and proliferation, resulting in hemolytic anemia and septicemia [7]. Without treatment, the disease has a high mortality, ranging between 40 and 88% cases [1]. Previous reports have demonstrated that 35% of all complications associated to Bartonellosis are of the infectious type [8]. Some patients go on to develop a chronic phase, characterized by a non-scarring, vascularized, verrucous skin lesion commonly known as the Peruvian Wart [1]. Children in endemic areas are more likely to develop these wart-like lesions and resolution occurs spontaneously but may take several weeks to months [1, 9].

Concomitant infections have been associated with the immunosuppression induced by the acute phase of the disease. The most common co-infections include *Salmonella typhi* and non-typhi, *Shigella dysenteriae*, *Staphylococcus aureus*, *Klebsiella* spp., toxoplasmosis reactivation, disseminated histoplasmosis, *Pneumocystis jiroveci* pneumonia, leptospirosis, and malaria due to *Plasmodium vivax* [5, 6]. However, there is no evidence reporting a molecularly diagnosed co-infection with *Mycobacterium tuberculosis* among patients immunosuppressed by the acute phase of Carrion’s disease [10, 11].

We report an outbreak of Bartonellosis in a coastal rural community in the Piura region, in which some patients had a coinfection with *Mycobacterium* spp. confirmed by genomic sequencing.

## Main text

### Methods

#### Study setting

This study was carried out between May and November of 2013 in the district of Lalaquiz, a province of Huancabamba with a population of 4793 and located in the Northern coast of Peru, 45 km south west from the city of Piura. The Lalaquiz district belongs to the Morropón-Chulucanas Medical Care Network, which has an integrated system of epidemiological surveillance and notification of emerging metaxenic diseases such as Carrión’s disease in the health facilities it manages.

Seventy venous blood samples of patients with clinical suspicion for Carrion’s disease were collected in the Regional Laboratory of health network Morropón-Chulucanas. Sampling is not probabilistic and all patients who met the inclusion criteria were considered. The inclusion criteria were patients who arrived at the outpatient clinics with acute, undifferentiated, febrile illness along with one or more of the following symptoms: headache, muscle pain, ocular and/or joint pain, nausea, vomiting, sore throat, cough, rhinorrhea, difficulty breathing, diarrhea, jaundice, generalized fatigue, cough, among others. The exclusion criteria included patients with an identifiable source of infection such as sinusitis, pneumonia, acute otitis media, acute upper tract infections, among others.

Samples were collected following a written informed consent signed by all participants above 18 years of age before enrollment. The written consents of underage patients, below 18 years of age, were signed by their parents or respective caregivers. All the laboratory results were given to the local physicians in a timely manner to continue the appropriate treatment. This study was approved by the Ethics Committees from the *Hospital Docente Regional de Cajamarca* in Peru.

#### Samples

A venous blood sample was collected per patient by using Vacuette^®^ EDTA blood collection tubes (Greiner Bio-One GmbH, Frickenhausen, Germany) and these were stored at 4 °C until processing. Molecular analysis was carried out as part of the febrile syndrome surveillance program conducted by the Regional Directorate of Health in the department of Piura.

#### Bacterial culture conditions

Microorganisms were cultured as previously described [12] with slight modifications. 200 µL of the blood sample were grown in Columbia blood agar medium (Oxoid Ltd, Basingstoke, Hants, United Kingdom) supplemented with 10% Sheep Blood defibrinated (Oxoid Ltd, Basingstoke, Hants, United Kingdom) and incubated at 28 °C for approximately 45 days under anaerobic conditions. Colonies were identified based on the characteristic morphology and gram staining (Sigma-Aldrich, St. Louis, MO, USA).

#### DNA extraction

The DNA was extracted from 200 µL of blood samples using High Pure Kit Preparation template (Roche Diagnostics GmbH, Mannheim, Germany). Bacterial DNA obtained after extraction was eluted in 100 µL of nuclease free water and then processed or stored at − 20 °C until use.

#### Amplification of a *Bartonella* spp. specific *16S rRNA* gene fragment

A 438 bp fragment of the *16S rRNA* gene specific for the genus *Bartonella* was amplified both in blood samples as previously described [13]. Amplified products were gel recovered, purified using SpinPrep™ Gel DNA Kit (EMD Biosciences, Madison, WI, USA) and sent to be sequenced (Macrogen Inc., Geumcheon-gu, Seoul, Korea).

#### Amplification of *16S rRNA* gene fragments using universal primers

For the case of *Mycobacterium*, molecular diagnosis was confirmed by amplification and sequencing of a 1503 bp region of the universal *16S rRNA* gene fragments using universal primers [14]. When no amplification product was obtained, *16S rRNA* gene-universal primers were used, and the product obtained was also sequenced.

#### Data analysis

DNA sequences were analyzed with the BLAST analysis tool and compared with the GenBank database.

### Results

A total of 70 (n = 70) samples with clinical criteria for acute phase of Bartonellosis and a positive peripheral blood smear were included. Of all the samples, 22.85% (n = 16) of cases were positive for *B. bacilliformis* by PCR and automatic sequencing. Of those positive samples, 62.5% (n = 10) cases were positive only for *B. bacilliformis* and 37.5% (n = 6) cases were positive to both *Mycobacterium* spp. and *B. bacilliformis*.

**Fig. 1:**
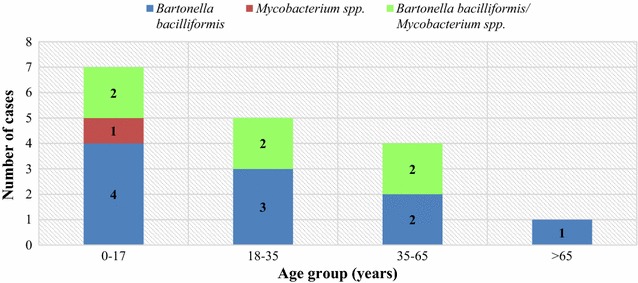
PCR results for 16S *Bartonella bacilliformis* and *Mycobacterium* spp. according to age group

The mean age of patients positive for *Bartonella* spp by PCR amplification of the 16S rRNA was 54.77 years (mean age of males = 31.6, mean age of females = 32.5), of which 50% (n = 8) cases were below 20 years of age. Of all the positive samples, 18.75% (n = 3) had a family history of a previous or current diagnosis of Carrion’s disease. Of these samples positive for *B. bacilliformis*, 81.25% (n = 13) cases were women and 18.75% (n = 3) cases were men (Fig. 1).

The majority of patients diagnosed with Carrion’s disease were from location of Tunal with 81.25% (n = 13) cases. The frequency of co-infections with *Mycobacterium* spp. was also the highest in this town (Table 1).

**Table 1:** Demographic characteristics of patient’s positive to *Bartonella bacilliformis* and *Mycobacterium* spp.

Characteristics general	Total patients n = 70 (%)	Only *Bartonella bacilliformis* n = 10 (%)	Only *Mycobacterium* spp. n = 1 (%)	*Coinfection Bartonella bacilliformis* and *Mycobacterium* spp. n = 6 (%)
Age				
0–17	20 (28.6)	4 (40.0)	1 (100.0)	2 (33.3)
18–35	19 (27.1)	3 (30.0)	0 (0.0)	2 (33.3)
35–65	24 (34.3)	2 (20.0)	0 (0.0)	2 (33.3)
> 65	6 (8.6)	1 (10.0)	0 (0.0)	0 (0.0)
Genero				
Female	41 (58.6)	8 (80.0)	1 (100.0)	5 (83.3)
Male	29 (41.4)	2 (20.0)	0 (0.0)	1 (16.7)
Location				
Tunal	25 (35.71)	7 (70.0)	1 (100.0)	5 (83.3)
Guayaquil Bajo	8 (11.4)	1 (10.0)	0 (0.0)	0 (0.0)
Maray Chico	1 (1.4)	0 (0.0)	0 (0.0)	1 (16.7)
Ambuñique	3 (4.3)	1 (10.0)	0 (0.0)	0 (0.0)
Maylan	1 (1.4)	1 (10.0)	0 (0.0)	0 (0.0)

The most common symptoms for patients with Bartonelosis were headaches, with a frequency of 70.0% (n = 7) cases followed by malaise with 60.0% (n = 6) cases. Similarly, these symptoms were also the most common experienced by the patient’s positive to both *B. bacilliformis* and *Mycobacterium* spp. (Table 2).

**Table 2:** Clinical features experienced by patient’s positive to *Bartonella bacilliformis* and *Mycobacterium* spp. by PCR amplification

Symptoms	Total patients n = 70 (%)	Only *Bartonella bacilliformis* n = 10 (%)	*Bartonella bacilliformis* and *Mycobacterium* spp. n = 6 (%)
Headache	40 (57.1)	7 (70.0)	3 (50.0)
Malaise	45 (64.3)	6 (60.0)	2 (33.3)
Arthralgia	21 (30.0)	4 (40.0)	1 (16.6)
Myalgia	18 (25.7)	3 (30.0)	1 (16.6)
Cough	8 (11.4)	2 (20.0)	0 (0.0)
Pallor	7 (10.0)	1 (10.0)	0 (0.0)
Diarrhea	7 (10.0)	1 (10.0)	0 (0.0)
Abdominal pain	5 (7.1)	1 (10.0)	1 (16.6)
Hyporexia	7 (10.0)	1 (10.0)	0 (0.0)
Jaundice	2 (2.9)	0 (0.0)	0 (0.0)

### Discussion

Carrion’s disease is associated to high mortality rates without prompt treatment, stressing the importance of early detection with efficacious diagnostic tools. Given that the pathogenesis includes a *Bartonella*-induced immunosuppressive state, this disease is also associated to many comorbidities and complications including co-infections with opportunistic microorganisms.

In this report, 22.8% of the samples tested positive for *Bartonella* spp. via PCR amplification of the 16S rRNA subunit. This molecular test has a sensitivity and specificity of 100% [12]. Genetic sequencing confirmed that the genome of all the positive samples belonged to *B. bacilliformis.* However, 77.2% of the samples that were interpreted as positive with a peripheral blood smear, were negative with PCR. Furthermore, authors reported a sensitivity of 36% during an outbreak in Urubamba in 1998 [15] and a sensitivity of 24% in Ancash [16]. On the other hand, the samples positive for *B. bacilliformis* were unable to grow in selective culture mediums. This could be explained due to the complex nutritional requirement and the high risk of contamination associated to this procedure attributing to its reported low sensitivity [17].

A transient immunosuppressive state has been described during the acute phase of the infection by *B. bacilliformis* [18]. Complete blood count may show a discrete lymphopenia associated with a significant decrease in TCD4+ lymphocytes with an inversion of the TCD4+/TCD8+ ratio [19]. It is during this period of immunosuppression that co-infections with some opportunistic microorganisms have been described. These infections include, *S. typhi* and *non typhi*, *S. dysenteriae*, *S. aureus*, *Klebsiella* spp., *Enterobacter* spp., *Candida* spp., *P. vivax*, among others [10, 11]. Mycobacterial infections have been described in patients with varying degrees of immunosuppression, especially in developing countries [20]. These infections may be caused by *M. tuberculosis* or other species of *Mycobacterium*.

In Peru, the incidence of tuberculosis is 119 per 100,000 people in the general population [20]. The primary infection of *M. tuberculosis* is controlled in 90% of adult patients, and it becomes latent. Reactivation occurs in immunosuppressed patients, this could occur due to HIV infections, malnutrition, chronic kidney disease, post-transplanted patient in treatment with immunosuppressants, among others [21]. In this outbreak, 6 patients (37.5%) were molecularly diagnosed with *Mycobacterium* spp. and *B. bacilliformis.*, which can be explained by the transient cellular immunosuppression during the acute phase of Carrion’s disease and points out the importance of a thorough physical examination and anamnesis in order to rule out a concomitant *Mycobacterium* spp. infection in endemic communities., which could have implications in the natural history of Carrion’s disease, treatment outcomes and patient morbimortality. The presentation of this coinfection is particularly important from the epidemiological point of view, since *M. tuberculosis* infections are very prevalent in Peru, therefore further studies are required.

Analysis of the samples obtained from the surveillance program for vector-borne diseases, indicate an outbreak of Carrion’s disease in a coastal community in the Piura region. There have been sporadic reports describing Bartonellosis in the Peruvian coastal communities such as Chincha, Ica, and Huaral [5] as well as some Ecuadorian communities such as, Manabi [22]. These series of reports may suggest a trend in which Carrion’s disease is spreading to coastal communities that did not previously have favorable environmental characteristics for vector proliferation.

One of the reasons that may explain this outbreak is the periodic climate shift that the Piura region cyclically experiences. Previous reports have described a direct correlation between Carrion’s disease transmission and the increase in ambient temperature and humidity. These climate variations have been reported to cycle every 4–8 years, depending on the presence of El Niño’s climate phenomenon, and are associated to an increase of cases in endemic regions and new cases in areas not previously reported [23]. Seasonality has also been associated to the incidence of infection with an initial increase during December and a peak in February and March [23]. This outbreak occurred during months of low incidence which may suggest that more cases of Bartonellosis could be confirmed if molecular tests such as PCR were applied to samples obtained during the months of high incidence.

In conclusion, Carrion’s disease is an emergent, forgotten disease with a high morbimortality if left untreated. The co-infection with *Mycobacterium* spp. found in some samples positive for *B. bacilliformis*, highlights the importance of considering other opportunistic infections with microorganisms not commonly described, such as *M. tuberculosis*. The incidence of pulmonary tuberculosis is high in developing countries, therefore the rate of co-infection with *B. bacilliformis* may be significantly higher in regions where the latter microorganism is endemic. Additionally, this outbreak demonstrates an increase in the geographic extension of Carrion’s disease to regions not previously described that may be associated to climate phenomenoms such as El Niño/Southern Oscillation (ENSO) and should shift the focus of new research on novel diagnostic methods, identification of alternative vectors and epidemiological management of outbreaks.

## Limitations

The sampled population is small and located in one particular region of Peru, and therefore results cannot be extrapolated to other areas endemic to *B. bacilliformis*. Furthermore, due to the nature of the febrile surveillance program, patient follow-was not possible after the initial management, creating gaps of knowledge regarding treatment success rates, complication rates and outcomes amongst co-infected patients. However, this study represents the first molecularly confirmed co-infection of *B. bacilliformis* and *Mycobacterium* spp. and may lead to new research in this field.

## Abbreviations

PCR: reaccion polimerasa chain
DNA: deoxyribonucleic acid
rRNA: ribosomal ribonucleic acid
bp: base pairs
